# Genomic data illuminates demography, genetic structure and selection of a popular dog breed

**DOI:** 10.1186/s12864-017-3933-x

**Published:** 2017-08-14

**Authors:** Pamela Wiener, Enrique Sánchez-Molano, Dylan N. Clements, John A. Woolliams, Marie J. Haskell, Sarah C. Blott

**Affiliations:** 1 Roslin Institute and Royal (Dick) School of Veterinary Studies, University of Edinburgh, Easter Bush, Scotland UK; 2 Scotland’s Rural College, Edinburgh, Scotland UK; 3 University of Nottingham, Sutton Bonington, England UK

**Keywords:** Genetic differentiation, Population structure, Dogs, Canine genetics, Craniofacial morphology

## Abstract

**Background:**

Genomic methods have proved to be important tools in the analysis of genetic diversity across the range of species and can be used to reveal processes underlying both short- and long-term evolutionary change. This study applied genomic methods to investigate population structure and inbreeding in a common UK dog breed, the Labrador Retriever.

**Results:**

We found substantial within-breed genetic differentiation, which was associated with the role of the dog (i.e. working, pet, show) and also with coat colour (i.e. black, yellow, brown). There was little evidence of geographical differentiation. Highly differentiated genomic regions contained genes and markers associated with skull shape, suggesting that at least some of the differentiation is related to human-imposed selection on this trait. We also found that the total length of homozygous segments (runs of homozygosity, ROHs) was highly correlated with inbreeding coefficient.

**Conclusions:**

This study demonstrates that high-density genomic data can be used to quantify genetic diversity and to decipher demographic and selection processes. Analysis of genetically differentiated regions in the UK Labrador Retriever population suggests the possibility of human-imposed selection on craniofacial characteristics. The high correlation between estimates of inbreeding from genomic and pedigree data for this breed demonstrates that genomic approaches can be used to quantify inbreeding levels in dogs, which will be particularly useful where pedigree information is missing.

**Electronic supplementary material:**

The online version of this article (doi:10.1186/s12864-017-3933-x) contains supplementary material, which is available to authorized users.

## Background

Over recent years, there has been increased concern about the genetic health of domesticated animal species and its relationship to levels of inbreeding and genetic diversity. This problem has been particularly pronounced in dogs, where founder effects at breed formation, extensive use of popular sires and intensive selection practices have had a negative impact on the genetic health of many breeds [1] and have contributed to the propagation of unfavourable traits [2] and hereditary diseases [3, 4]. Concerns about these issues have led to recent efforts to improve genetic health in this species [5–7]; a key component of this process involves characterisation of genetic diversity, structure and inbreeding within dog breeds.

The Labrador Retriever derives from working dogs of the Labrador/Newfoundland region of Canada that were brought in the nineteenth century to Britain by aristocrats and used as retrievers for hunting. Following an association between the Earl of Malmesbury and Duke of Buccleuch, a breeding program was established at the Scottish Buccleuch Kennels in the 1880s, which contributed to the foundation of the modern Labrador Retriever breed. The breed was formally recognized by the Kennel Club in 1903 (and in 1917 by the American Kennel Club). Labrador Retrievers are noted for their retrieving ability but also for their easy temperament, making them popular family pets. It is by a large margin the most populous Kennel Club-registered breed in the UK (32,507 new registrations in 2015, compared to 22,577 for the next most common breed, Cocker Spaniel). Labrador Retrievers are also the most popular breed in many other developed countries, including the U.S.A., Australia, Canada, and Sweden. Labrador Retrievers are predisposed to a large number of heritable disorders [3, 4], including hip and elbow dysplasia, debilitating orthopaedic diseases [8].

While traditional pedigree-based methods can be used to characterise breed attributes, the development of molecular genetic tools has provided additional resources with the potential for finer-scale analysis. While the uptake of molecular methods was initially slower in companion animals than in livestock, high-density single nucleotide polymorphism (SNP) arrays are available for the major companion animal species (dogs, cats, rabbits), providing a valuable tool for genetic diversity characterisation and for the inference of demographic and selection processes. If the costs of high-throughput sequencing continue to fall, this strategy may become the preferred alternative to commercial SNP arrays. The aim of this study was to apply high-density SNP data to elucidate population-level processes in the UK Labrador Retriever population.

## Methods

### Samples

The animals in the study were Kennel Club-registered UK Labrador Retrievers born between 2002 and 2008. The samples were collected as part of a project on canine hip dysplasia [9, 10]; all dogs had previously been radiographed under the British Veterinary Association (BVA)/Kennel Club (KC) hip scoring scheme [11].

### Genotypes

1008 DNA samples were genotyped using the Illumina canine high density beadchip containing 173,662 SNPs. Quality control was applied to assure both sample and marker quality. A sample call rate threshold of 95% was applied, removing 272 samples with low call rate and 8 females misidentified as males by Illumina Genome Studio software (i.e. with low heterozygosity on the X chromosome), leaving 728 animals in the final sample (195 males, 533 females). Initial marker quality criteria were applied using Genome Studio. A total of 59,260 markers were discarded for low call rate (<98%), low reproducibility (GTS < 0.6), low or confounded signal (ABR mean < 0.3) and low minor allele frequency (MAF < 0.01). Further quality control on the markers was applied using PLINK [12], removing SNPs on the sex chromosomes and those showing deviation from Hardy-Weinberg equilibrium (*p* < 8.0E-8, applying a Bonferroni correction), leaving 117,971 SNPs for further analysis.

### Phenotypes

Phenotypic and lifestyle information was collected from dog owners using a questionnaire (Additional file 1). The classification (role) of dogs as gundog, pet, showdog or “other work” was based on two questions: the first related to whether it was a working dog (options: pet, gundog, guide dog, sniffer dog, hearing dog or other work) and the second related to the activities in which the dog participates (options: none, agility trials, showing, field trials, obedience trials or other). Dogs that were identified as guide dogs, sniffer dogs, hearing dogs or other work were pooled together as “other work.” A strict quality control procedure was implemented such that any dogs with questionable or uninterpretable information (e.g. dogs identified as both “gundog” and “pet”) were considered to have unknown classification. Dogs with rare coat colours (black & tan, fox red, liver) were considered unclassified, leaving three categories: black, yellow and chocolate. Geographical information was summarized according to region of the UK: 1 = Orkney Islands, 2 = Scotland (other than Orkney), 3 = northwest England, 4 = northeast England, 5 = Wales, 6 = Midlands (England), 7 = East Anglia (England), 8 = southwest England, 9 = southeast England, 10 = Northern Ireland.

### Pedigree analysis and estimation of effective population size

In order to assess whether the sample of 728 was representative of the UK Labrador Retriever population, we performed a preliminary analysis of the pedigree relationships. The mean kinship coefficient (half the relationship coefficient) [13, 14] for the 728 analysed dogs was 0.038 (se 6.20e-05), similar to the level for a random set of 728 Labrador Retrievers of similar ages selected from the full pedigree (0.030, se 4.73e-05) (the mean kinship coefficient between the experimental set and the random set was 0.033, se 3.77e-05). Thus we conclude that the dataset reflects the overall diversity in the breed. The skewed sex ratio of the sample reflects the population of dogs on which hip scoring is performed [15], which may be due to the greater proportion of females used for breeding (there is no evidence for a sex ratio bias in the breed as a whole [16]).

Analysis of pedigree-based inbreeding was performed on a pedigree of 25,526 animals including the genotyped animals. Calculations of inbreeding coefficients were conducted using ENDOG software [17]. The following parameters describe the completeness of the pedigree: mean maximum generations (number of generations separating the individual from its furthest ancestor) = 11.26, mean complete generations (number of generations, *g*, separating the individual from its furthest ancestor, where all 2^*g*^ ancestors of the individual are known, = 3.58, mean equivalent generations (sum of (1/2)^*n*^ terms over all known ancestors, where *n* is the number of generations separating the individual from the ancestor) = 5.58.

The inbreeding effective population size (*N*_*e,*_ which predicts the rate of decrease in heterozygosity) was estimated as *1/(2 ∆F L)* where *F* is the inbreeding coefficient, *∆F* is the annual rate of inbreeding and *L* is the average generation interval (estimated as 4.5 years for the UK Labrador Retriever population [18]), such that *∆F L* is the rate of inbreeding per generation [13]. We then regressed the natural logarithm of *(1-F)* on birthdate, such that *∆F L* was estimated by (−1×) the slope of the relationship between *ln(1-F)* and birthdate [19]. Birthdate was available for 17,348 individuals in the pedigree.

### Population structure analyses

Principal component analysis (PCA) was performed on the full dataset using GCTA [20]. Structure version 2.3.4 [21] was applied to approximately one third of the genotype data (every third marker across the genome, resulting in 39,324 markers), to reduce linkage disequilibrium between markers and for computational ease. An alternative data subset was generated using linkage disequilibrium-based data pruning in PLINK [12], with the default options (window size = 50 SNPs, the number of SNPs to shift the window at each step = 5, the variance inflation factor threshold = 2), which generated a data subset of 13,310 SNPs. Five replicates were run under the admixture model for each value of K (number of clusters) from 1 to 5 with a burn-in period of 20,000 iterations and 10,000 iterations after burn-in. Individuals were labelled according to role of the dog (gundog, pet, showdog, other work, unknown), geographical location (1–10) and coat colour (black, yellow, chocolate) to assess whether these factors were associated with genetic clusters. Delta K, which estimates the second derivative of the log likelihood profile from the Structure analysis, was calculated for K = 2 to 4 [22]. Results were graphed using *Distruct* [23].

### Linkage disequilibrium calculation and estimation of effective population size

Linkage disequilibrium, measured as the squared correlation between SNP pairs (*r* ^*2*^), was calculated using PLINK [12] for all syntenic marker pairs on the autosomes. To enable plotting LD as a function of marker distance, marker pairs were divided into distance bins and *r* ^*2*^ was averaged for each bin.

The expression *E(r* ^*2*^ *) = 1/(α + 4N*_*e*_ *c)* was used to estimate effective population size (*N*_*e*_), based on the relationship between LD and marker distance [24], using the approach introduced by references [25] and [26]; *c* is the recombination frequency (expressed as distance in Morgans) and *α* is set equal to 1 when mutation is not considered [24] and to 2 when mutation is accounted for [27]. Distance between markers in Morgans was estimated based on 0.97 cM/Mb [28]. Average *r* ^*2*^ values between markers with minor allele frequency > 0.1 were calculated for each *c* value/distance bin and *N*_*e*_ values were estimated for each bin using two values of *α* (1 or 2). *N*_*e*_ values were also estimated based on *r* ^*2*^ adjusted for sample size (adjusted *r* ^*2*^ *= r* ^*2*^ *–1/2n*, where *n* = 728) [29]. Assuming linear population growth over time, each bin is associated with the *N*_*e*_ of a specific time in the past, with the number of generations in the past given by *1/2c*.

### Runs of homozygosity

Runs of homozygosity (ROH) in the full dataset were identified using PLINK [12] using the default options. This algorithm scans SNP data along the genome for homozygous stretches. For each SNP, it calculates the proportion of completely homozygous windows that encompass that SNP. If more than a specified proportion (default = 5%) of these windows are completely homozygous, then the SNP will be included in the putative ROH. If the putative ROH includes more than a specified threshold (default = 100) of such consecutive SNPs, it is declared a ROH. The algorithm allows for a specified number of heterozygous (default = 1) and missing SNPs (default = 5) within a ROH. ROHs were restricted to include at least 100 SNPs and be over 1 Mb in length. The “--homozyg-group” option was used for a subset of chromosomes to obtain pools of overlapping segments that share alleles. F_ROH_ was defined as the total length of ROHs in the genome divided by 2,198,710,490, which is the number of base pairs covered by the CanineHD chip, according to CanFam2.0. Basic F_ROH_ statistics were calculated for the full dataset and for subgroups. *χ* ^*2*^ tests were used to test for association between cluster assignment and a subset of overlapping ROH segments.

### Population differentiation analysis

For each SNP marker across the genome, unbiased estimates of Weir and Cockerham’s F_ST_ [30] were calculated as functions of variance components [31] to compare individuals from each of two sub-populations defined by the Structure analysis. A similar approach has been applied previously to identify genomic regions showing differentiation between breeds [32, 33] but in this study, all individuals were members of the same breed. In order to maximize our ability to identify regions associated with the within-breed cluster division, we thus chose to analyse a subset of the most highly differentiated individuals (27 from each of the two clusters), as defined by Structure.

To reduce random effects of individual SNPs with extreme allele frequency differences, individual F_ST_ values were averaged across sliding windows. In order to determine how best to define these windows (i.e. by physical size or number of SNPs), we compared the distribution of numbers of SNPs per window of fixed physical size to the distribution of physical sizes per window of fixed number of SNPs, where an odd number of SNPs was used to give equal numbers of markers on either side of the central SNP and the choice of numbers of SNPs per window (9, 13 and 17) was based on previous studies using high-density SNP panels [33, 34]. The distribution of physical sizes per window of fixed number of SNPs resulted in far less dispersed distributions (Additional file 2) in contrast to studies conducted using less dense marker panels [35], thus individual F_ST_ values were averaged across sliding windows of 9, 13 and 17 SNPs (F_ST_-windows-9, F_ST_-windows-13, F_ST_-windows-17). For each window size, the top 0.05% of F_ST_ windows were identified as differentiated regions [33, 34]. Markers within 1-Mb in the set of top F_ST_ windows were grouped together in regions.

LiftOver [36] was used to convert SNP positions from CanFam2.0 to CanFam3.1. All annotated genes within regions showing strong evidence for differentiation were extracted from the CanFam3.1 assembly in Ensembl [37] using BioMart [38]. Annotated genes found within 0.5 Mb of the highly differentiated regions were evaluated for known functions or disease associations. Gene ontology (GO) enrichment of biological processes was evaluated using the PANTHER Overrepresentation Test in which the target list of genes was compared to the *Canis familiaris* reference list [39].

## Results

### Population structure

**Fig. 1:**
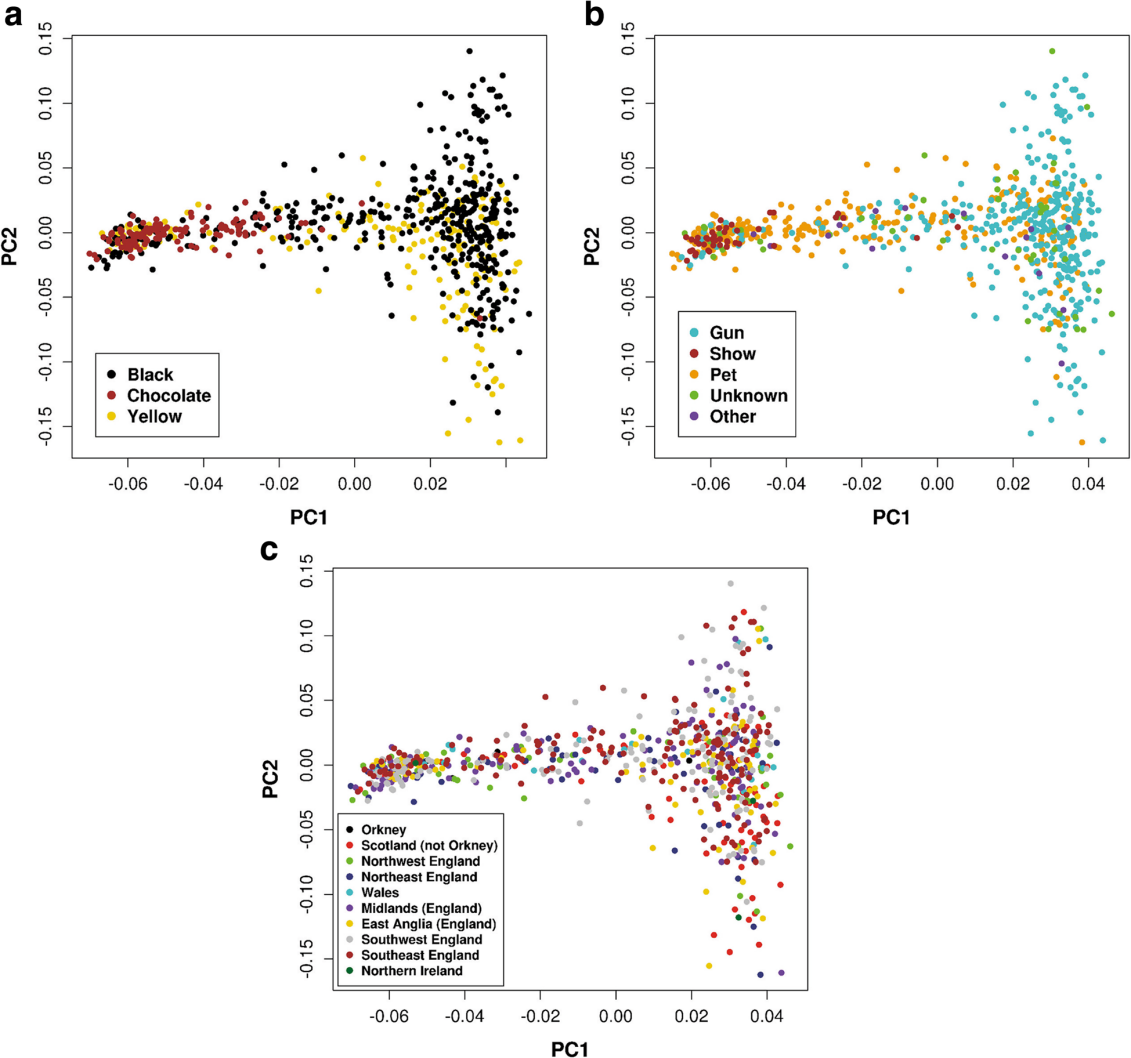
Principal components analysis (PCA) of UK Labrador Retrievers: **a** dogs labelled by coat colour (*black, yellow, chocolate*); **b** dogs labelled by role (gundog, showdog, pet, unknown, other); **c** dogs labelled by geographical location

In order to characterise the structure of the UK Labrador Retriever population, Principal Components and Structure analyses were performed. The first 250 principal components (PCs) from the PCA of the genotype data, chosen according to Kaiser’s criterion of inclusion of all components with eigenvalues greater than one [40], explained approximately 65% of the total variance, with the first, second and third PCs explaining approximately 6.8%, 0.94% and 0.82% of the total variance (Fig. 1). From a visual assessment, the first principal component mainly separated showdogs from gundogs, where gundogs generally had higher PC1 values although there was some overlap (Fig. 1a). Dogs classified as unknown or “other work” were distributed across the PC1 spectrum; the majority of pets were found clustered with showdogs although a sizeable minority clustered with gundogs (~38% had PC1 values greater than 0). The dogs with chocolate coats had negative values on the PC1 axis and were thus found in the region of the PCA plot occupied by the showdogs; yellow and black dogs were distributed across PC1 (Fig. 1b). There was no obvious geographical structuring (Fig. 1c). Discrimination of individuals on PC2 was related to different sire families (results not shown); there was also separation between yellow and black dogs such that almost all individuals with positive PC2 values were black.

**Fig. 2:**
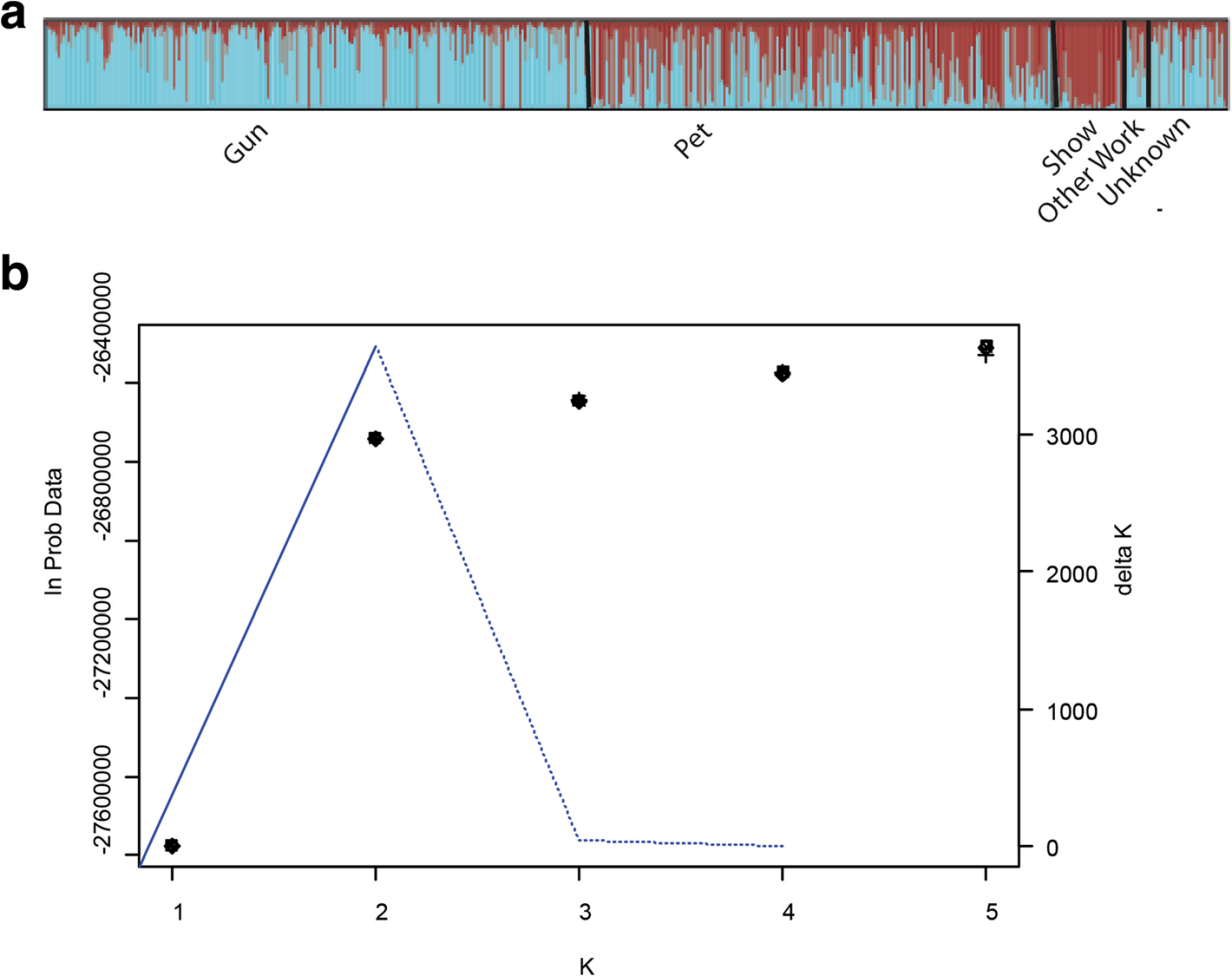
Results from Structure analysis of UK Labrador Retrievers: **a** assignment probabilities of dogs from different role categories to clusters identified for K = 2; **b** plot of ln(Probability of Data) (5 points per K value) and delta K value (*dotted blue line*) [22] as a function of K value

The Structure analysis of one-third of the markers showed the same clustering results as those for the LD-pruned markers, thus we describe the former below. The log likelihood profile increased from K = 1 to 5, but was fairly flat for K ≥ 2 and delta K was maximized at K = 2 (Fig. 2), together suggesting that the best estimate for number of clusters was K = 2. The overall allele frequency divergence between the two clusters was 0.0491. Of the 728 dogs, 361 (50%) had high assignment probability (>0.8) to cluster 1, while 168 (23%) had high assignment probability to cluster 2. The remaining 199 dogs (27%) had intermediate assignment probabilities (0.2–0.8) to both clusters. Results were highly consistent with those from the PCA: across the 728 dogs, the correlation between PC1 and assignment probability to cluster 1 was almost identical to 1. Proportion of membership of the pre-defined populations (q) to the two clusters (K = 2) measures the average assignment probability to a cluster across all individuals in that population (Table 1). Gundogs had high membership coefficients for cluster 1 (q = 0.827) while showdogs had high membership coefficients for cluster 2 (q = 0.896). Pets had a slightly higher membership coefficient for cluster 2 (q = 0.552). Dogs classified as “other work” or unknown had higher membership coefficients for cluster 1 than 2. Yellow and black dogs had higher membership coefficients for cluster 1 (q = 0.666 and 0.723, respectively) while chocolate dogs were more strongly associated with cluster 2 (q = 0.839). Dogs from all regions except Orkney had higher membership coefficients for cluster 1 (q = 0.521–0.721), where the highest value was for dogs from mainland Scotland. Dogs from Orkney (only four dogs) had a higher membership coefficient for cluster 2 (q = 0.643). These results were consistent with the PCA in which three of the four Orkney dogs had PC1 values less than −0.030 and ~50% of dogs from mainland Scotland had PC1 values greater than 0.028 (~82% with PC1 values >0).

**Table 1:** Average membership coefficients to cluster 1 for different classification categories (K = 2 Structure analysis)

Category	Average membership coefficient to cluster 1
Role/Activity	
Gun	0.827
Pet	0.448
Show	0.104
Other	0.631
Unknown	0.644
Coat Colour	
Black	0.723
Yellow	0.666
Chocolate	0.161
Geographical location	
Orkney	0.357
Scotland (not Orkney)	0.721
NW England	0.521
NE England	0.553
Wales	0.609
Midland	0.557
East Anglia	0.665
SW England	0.625
SE England	0.654
Northern Ireland	0.680

In the K = 3 analyses, cluster 1 from the K = 2 analyses was split into two new clusters, for which showdogs had low average membership coefficients (q = 0.076 and 0.033). The average membership coefficients of yellow and black dogs for the two clusters were similar (yellow: 0.298 and black: 0.259 for one cluster, yellow: 0.381 and black: 0.476 for the other cluster) while the average membership coefficients of chocolate dogs for both clusters were lower (0.060 and 0.107).

### Pedigree-based estimates of effective population size

The relationship between the annual rate of inbreeding and time (birthdate) was used to estimate the inbreeding effective population size, based on the UK Labrador Retriever pedigree. Assuming a generation interval of 4.5 years and using the entire pedigree with birthdates, the estimate of *N*_*e*_ was 55. Two modifications to the dataset were made to assess effects on the estimated *N*_*e*_. If the oldest dogs in the pedigree (birthdates prior to 19/02/1982, 30,000 days since 01/01/1900, approximately 14% of the population) were removed, the estimate was 66. If individuals with inbreeding coefficient equal to 0 (indicating lack of relevant pedigree information, such that mean complete generations, equivalent generations and maximum generations were all significantly lower for the individuals with F = 0) are removed, the estimate of *N*_*e*_ was 82.

### Linkage disequilibrium and effective population size

**Fig. 3:**
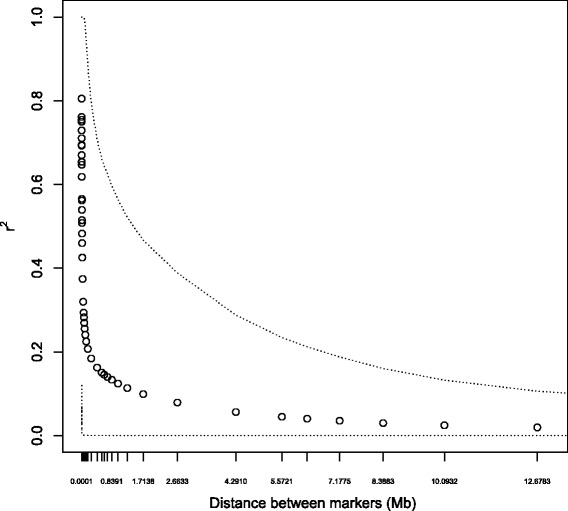
Pattern of linkage disequilibrium as a function of marker distance for UK Labrador Retrievers: points represent average *r* ^2^ for pairs of markers in bins of specified average between-marker distance. *Dotted lines* indicate the 2.5th and 97.5th percentiles of the *r* ^2^ distribution (i.e. where 95% of the data points are located)

For comparison with the pedigree-based estimates, high-density SNP data was also used to estimate *N*_*e*_, based on the pattern of pairwise LD as a function of marker distances. LD shows the typical decreasing exponential relationship with marker distance (Fig. 3), with average *r* ^2^ equal to 0.625 for bins with markers less than 1 kbp apart and an average *r* ^2^ less than 0.1 for bins with markers greater than 820 Kbp apart. Using average *r* ^2^ for markers between 33 and 100 cM apart (average physical distance ~41 Mb) provides an *N*_*e*_ estimate for ~1 generation in the past; estimates ranged from 74 to 88, depending on whether mutation is accounted for and whether *r* ^2^ was adjusted for sample size.

### Inbreeding and ROH

**Fig. 4:**
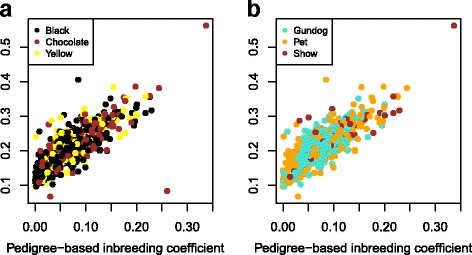
Relationship between pedigree-based (F_pedigree_) and genomic-based (F_ROH_) inbreeding coefficients for UK Labrador Retrievers, details in the main text: **a** dogs labelled by coat colour (*black, yellow, chocolate*); **b** dogs labelled by role (gundog, showdog, pet; dogs labelled as “unknown” or “other” are not shown on the plot)

Pedigree-based inbreeding coefficients were compared with ROH characteristics for the population. Pedigree-based inbreeding coefficients for the population ranged from 0.0000795–0.337 (mean = 0.0702) (Fig. 4). The number of ROH segments ranged from 31 to 104 (mean = 70.85) and the total length of ROH segments ranged from 147,273–1,236,070 kb (mean = 457,465). F_ROH_ ranged from 0.067 to 0.56 (mean = 0.21, se = 7.39e-05). The Pearson correlation coefficient between the pedigree-based inbreeding coefficient (F_pedigree_) and F_ROH_ (or total length of ROH segments) was 0.78 (0.77 when the individual with maximum inbreeding coefficient and total length of ROH segments was removed) (Fig. 4) while the correlation between number and total length of ROH segments was 0.79 and that between F_pedigree_ and number of ROH segments was 0.57 (with or without the individual with maximum inbreeding coefficient). The slope of the regression of F_ROH_ onto F_pedigree_ was 0.94 (s.e. 0.028). The characteristics of F_ROH_ within the role and coat colour groups were generally similar to those for the full dataset, although chocolate dogs and showdogs were somewhat distinct and both had higher values of F_ROH_ than the other groups. Mean values of F_ROH_ for the three coat colour groups were the following: 0.20 (se 1.2e-04) for black, 0.23 (5.7e-04) for chocolate and 0.21 (2.8e-04) for yellow; for the three role groups: 0.20 (1.3 e-04) for gundogs, 0.21 (2.0 e-04) for pets and 0.26 (1.6 e-04) for showdogs. The correlation coefficients between F_pedigree_ and F_ROH_ for the three coat colour groups were the following: 0.79 (black), 0.74 (chocolate) and 0.77 (yellow); for the three role groups: 0.76 (gundogs), 0.81 (pets) and 0.84 (showdogs). The slopes of the regression of F_ROH_ onto F_pedigree_ for the three coat colour groups were the following: 0.96 (se 0.036) for black, 0.89 (0.075) for chocolate and 0.97 (0.059) for yellow; for the three role groups: 0.99 (0.046) for gundogs, 0.97 (0.041) for pets and 0.87 (0.086) for showdogs.

The longest ROH in the genome (~115 Mb) was found on CFA1 in the most inbred individual (a female, chocolate showdog) in the study. ROHs covered by >400 (55%) individuals were found on CFA1 and CFA11. The ROHs that were shared by the largest number of dogs (581) were defined by SNPs on CFA11 (CFA11:42,380,188–42,480,533), a region that does not include any annotated genes (the closest gene is *ELAVL*2, which encodes a neural-specific RNA-binding protein). Other regions of high ROH coverage (>300 dogs, 41%) were found on CFA5, 24, 25, 32 and 35.

### Genomic differentiation between genetic clusters

We identified regions of the genome showing the greatest levels of differentiation (based on F_ST_) between the groupings highlighted in the population structure analyses. Having identified that K = 2 was the best estimate of cluster number, individuals were chosen for the differentiation analysis based on their membership coefficient for the two clusters from the K = 2 Structure analysis. Individuals were initially selected with extreme membership coefficients (> 0.90) and that were classified as either showdog or gundog; half-siblings were then removed, which left 115 “Cluster 1” dogs (gundogs) and 27 “Cluster 2” dogs (showdogs). In order to fairly compare Cluster 1 and Cluster 2, 27 Cluster 1 dogs with the highest membership coefficients were selected for differentiation analysis (average membership coefficient for cluster 1 = 0.996, for cluster 2 = 0.972).

The top 0.05% of F_ST_-windows (over all three window sizes) encompassed 20 regions on 12 chromosomes (Table 2). There were more regions identified for F_ST_-window-9 than the other two window sizes. The regions identified for the larger window sizes were generally a subset of those identified for F_ST_-windows-9 (the exceptions were CFA6:24.56 Mb, CFA13:4.88–5.08 Mb and CFA17:25.80 Mb, which were not seen in the F_ST_-windows-9 analysis). The maximum F_ST_-windows values for each window size were identified as the top 24 values: F_ST_-windows-9 values >0.700, F_ST_-windows-13 > 0.619, F_ST_-windows values-17 > 0.602. Protein-coding genes found within 0.5 Mb of the highly differentiated regions are shown in Additional file 3. In the GO enrichment analysis, none of the biological processes reached significance after correction for multiple testing due to the large number of background genes tested; however, the process with the lowest *p*-value was “regulation of anatomical structure morphogenesis” (*p* = 1.79e-04), which showed ~6-fold enrichment.

**Table 2:**
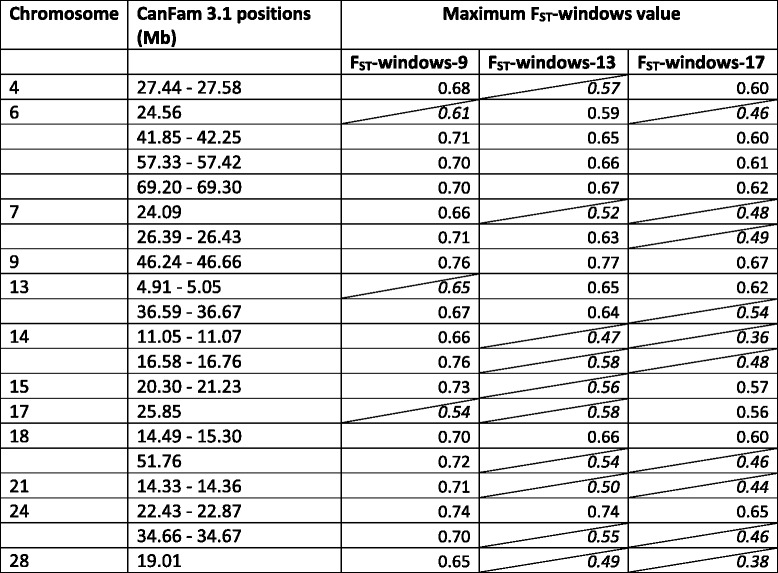
Genomic regions showing the highest levels of differentiation (top 0.05% of F_ST_-windows-9, −13 and −17) between the two clusters identified by Structure (K = 2): for each region including markers in the upper tail of the respective F_ST_-windows distribution, the maximum of F_ST_-windows values for the region is presented (italicized values in cells with diagonal lines indicate that the region did *not* feature in the extreme of the F_ST_-windows distribution for that window size). Regions were defined to incorporate neighbouring markers within 1-Mb distance

The total length of ROHs was greater for the 27 Cluster 2 dogs (mean = 615,062) than the 27 Cluster 1 dogs (mean = 532,892) described above (*p* = 0.009). This difference was still significant (*p* = 0.002) when the most highly inbred dog (a Cluster 2 dog, extreme value on Fig. 4) was removed from the analysis.

There was not a strong correspondence between the differentiated regions and the ROHs. However, five of the 20 highly differentiated regions coincided with 12 “overlapping” ROHs, those found in multiple individuals (where the overlap involved >2 SNPs). For all 12 of these overlapping segments, there was a significant association between cluster membership (defined by membership coefficient to cluster 1: “low” refers to ≤0.5, “high” refers to >0.5) and ROH grouping, as defined by the alleles at individual SNPs (*p* < 0.001).

## Discussion

### Within-breed genetic differentiation

An important tool in the genetic characterization of domesticated animal species is the application of statistical methods that group individuals into clusters without prior population labelling. In most cases, studies have demonstrated good correspondence between breeds and genetically-defined populations such that use of this approach can be particularly useful for identifying animals that do not fit the general genetic profile of a given breed, for example, cross-bred or misclassified individuals [41]. In some cases, however, clustering techniques have revealed population structure below the breed level, such as that seen in our analysis of the UK Labrador Retriever population, where both the Structure and PCA analyses indicated genetic subdivision into two groups. These groups were associated with the role of the dog: working (“field”, “gun”) dogs and show (“conformation”) dogs, while pets were a mixture of both types. There was also genetic differentiation between chocolate (brown) and both yellow and black dogs, with chocolate dogs primarily found in the showdog cluster. The proportion of variance explained by the first principal component (6.8%), which was strongly associated with the role factor, is similar to the level seen in clustering of geographically-related human populations (e.g. populations from the Middle East and East Asia [42]); its moderately low value indicates that while there is clear differentiation within the breed, a large proportion of the genetic variation is accounted for by unknown factors.

Within-breed differentiation has been previously demonstrated in livestock species, including the British Saddleback pig breed [43], Italian autochthonous donkeys [44], southwestern European goats [45] and British chicken breeds [41]. Several studies in dogs have also identified within-breed differentiation, which appears to derive from several sources. A similar case to the Labrador Retriever is the Border Collie [46], where working dogs were shown to be genetically differentiated from showdogs. However, most cases of within-dog-breed differentiation appear to be associated with geographical distance (unlike our study) or clear physical characteristics. Quignon et al. [47] analysed American and European samples from four breeds and demonstrated a clear genetic separation of US and EU Golden Retrievers. Björnerfeldt et al. [48] identified strong genetic differentiation in poodles due to size and coat colour. Standard poodles were clearly genetically distinct from all other poodles, while the smaller poodles were differentiated from each other based on a combination of size and coat colour. A study on Schnauzer breeds revealed a similar pattern of differentiation [49] such that Giant Schnauzers were strongly differentiated from the other Schnauzer breeds, while the smaller Schnauzers clustered based on both coat colour and size. Mellanby et al. [50] also demonstrated genetic structure within UK Cavalier King Charles spaniels, although the source of the differentiation was not clear.

### Highly-differentiated genomic regions

The F_ST_ analysis identified several genomic regions that were strongly genetically differentiated between the two Labrador Retriever clusters. As these clusters were associated with both coat colour and classification/role of the dog (i.e. gundog, showdog), we investigated the differentiated regions for the presence of genes that could be related to these groupings. The regions did not include either of the two genes known to determine coat colour in Labrador Retrievers (*TYRP1*, CFA9:12,685,439–12,710,290, and *MC1R*, CFA16: 89,912,119–89,920,977), suggesting that the stratification has not primarily been driven by selection for coat colour.

Regarding the role groups, there are morphological features that are known to differ between gundogs and showdogs. Showdogs tend to have heavier builds, with shorter legs compared to gundogs. In terms of head shape, showdogs tend to have shorter muzzles and slightly wider heads [51, 52]. The 20 differentiated regions did not include any of the six genes that have been associated with body size in between-dog-breed studies [53]. The regions did, however, include several genes that have been associated with craniofacial development and this finding was further supported by the results of the GO analysis of biological processes in which “anatomical structure morphogenesis” showed the strongest evidence of enrichment in the differentiated regions. Furthermore, two of the regions, on chromosomes 9 and 24, were located within 1 Mb of SNPs previously associated with canine skull shape in a between-breed GWAS study [54], suggesting that the genetic differentiation between the clusters may in part be related to this phenotype.

The CFA9 region is of particular interest: it overlaps an orthologous chromosomal region on human chromosome 17p in which deletions of various sizes are associated with Miller-Dieker syndrome (MDS), a congenital malformation associated with brain and craniofacial disorders (including lissencephaly, “smooth brain”) [55]. Deletions involving several of the highly differentiated genes in this region have been suggested to contribute to MDS, including *PAFAH1B1* (the primary lissencephaly-related gene, also known as *LIS1*, CFA9:46,647,994–46,727,422), *MNT* (CFA9:46,466,709–46,482,614) and *SMG6* (CFA9:46,161,531–46,405,748) [56–58]. In addition, the *HIC1* gene, located just outside the differentiated region (CFA9:46,159,464–46,162,004), functions as a growth regulator and has also been associated with MDS and craniofacial development. *HIC1*-deficient mice were shown to carry developmental abnormalities, including several craniofacial defects, acrania (partial or complete absence of the flat bones in the cranial vault), exencephaly (brain located outside of the skull) and underdeveloped ear [59]. *HIC1* was also strongly associated with cleft palate in a genome-wide association study of humans [60]. Other genes in the differentiated regions include *ALX3* (CFA6:41,903,464–41,906,963) and *CDK14* (CFA14:16,440,408–17,007,553), both of which have been associated with craniofacial development [61, 62].

Behavioural characteristics have also been shown to differ between gundogs and showdogs in the Labrador Retriever breed [63, 64]. While some of the genes in the differentiated regions are associated with neuronal or neurological function, we are not aware of any associations with behavioural traits. However, as the genetics of behaviour is still in its early stages and there is little evidence to definitively connect specific genes to these traits, we cannot conclude that these regions do not influence dog behavioural characteristics.

Only five of the 20 highly differentiated regions coincided with overlapping ROHs that were shared across individuals, suggesting that selection for cluster-related phenotypes is not strongly associated with extended homozygosity in the genome. However, for the overlapping ROHs that did coincide with differentiation regions, there was a significant association between cluster membership and ROH grouping, as defined by the alleles at individual SNPs, so it may be the case that some ROHs are related to selection for cluster-related phenotypes.

### Genomic characterisation of inbreeding

This study demonstrates the great potential for using genomic data to estimate inbreeding levels in domesticated animal species. The concept of using regions of homozygosity to estimate inbreeding levels dates back to 1999 [65] and it was first applied in humans [66, 67]. This approach has been applied to livestock in numerous studies [68–73] but only recently in dogs [74, 75]. Other genomic approaches to estimation of inbreeding have been implemented (e.g. diagonal elements of the genomic relationship matrix) but the ROH approach appears to be less influenced by allele frequencies and thus more accurate [68, 76]. Our analysis revealed a high correlation between F_pedigree_ and F_ROH_ (0.78), while some studies in cattle and pigs have shown lower correlations, for example, 0.015 in Brazilian Landrace pigs and 0.24 in Brazilian Large White pigs [69]; 0.47 in Danish Jersey cattle and 0.49 in Danish Red Cattle [68]. These correlations could have been influenced by the quality of either or both the pedigree- and genomic-based estimates and may have been influenced by the effective population sizes and/or depth of pedigree of the studied populations. Further studies in dogs will be required to develop the optimal approach(es) for estimating inbreeding based on ROH data, which may be breed-specific (i.e. influenced by the age and level of inbreeding within individual breeds). Considerations include specification of the parameters to use in defining ROHs as well as determination of the most useful range of ROH lengths to consider in the calculation of F_ROH_.

Both pedigree- and genomic-based estimates of effective population size were somewhat lower than those published previously for Labrador Retrievers. Our pedigree-based estimate for the full dataset (54.5) was lower than that published by the KC (81.7, for the period 1980–2014 [18]), although almost identical (82.3) when animals with F = 0 were removed; it was also considerably lower than an earlier pedigree-based estimate (114) [77]. The range of genomic estimates (74–88) was also highly consistent with the KC estimate. Thus a genomic approach to estimation of effective population size shows great promise, especially in situations where the depth of pedigree is low and thus pedigree-based estimates are likely to be poor.

## Conclusions

This study demonstrated that the UK Labrador Retriever population showed evidence of genetic stratification into two groups, one of which was primarily associated with working dogs and the other with showdogs; pet dogs were a mixture of both types. Genetic differentiation was also seen between the three main coat colour types, with chocolate dogs primarily associated with the showdog grouping. Identification of the genomic regions showing the greatest differentiation between the two sub-populations provided evidence that this stratification is related to morphological differences between showdogs and working dogs. Specifically, the differentiated regions included several genes associated with craniofacial development, which may contribute to the differences in head shape between the two groups. This study also found that the total length of homozygous segments (runs of homozygosity, ROHs) was highly correlated with the pedigree-based inbreeding coefficient.

## Additional files

Additional file 1: Questionnaire used to determine phenotypic and lifestyle information. (PDF 394 kb)

Additional file 2: Figure S1. a. Distributions of physical size for windows of fixed number of SNPs (9, 13, 17). b. Distributions of number of SNPs for windows of fixed physical size (150Kb, 225Kb, 300Kb; approximate genome-wide average sizes of 9-SNP, 13-SNP and 17-SNP windows). (PPTX 117 kb)

Additional file 3: Annotation of highly-differentiated regions: description of protein-coding genes found within 0.5 Mb of highly-differentiated regions (Table 2). (XLSX 19 kb)

## Abbreviations

ABR: Normalized intensity of heterozygote cluster
*ALX3*: Aristaless-like homeobox 3
BVA: British Veterinary Association
*CDK14*: Cyclin Dependent Kinase 14
CFA: *Canis familiaris*
cM: centiMorgans
GO: Gene ontogeny
GTS: GenTrain score
*HIC1*: Hypermethylated in cancer 1
KC: Kennel Club
LD: Linkage disequilibrium
*LIS1*: Lissencephaly 1
Mb: Megabase
*MC1R*: Melanocortin 1 receptor
MDS: Miller-Dieker syndrome
*MNT*: MAX Network Transcriptional Repressor
*N*_*e*_: Effective population size
*PAFAH1B1*: Platelet Activating Factor Acetylhydrolase 1b Regulatory Subunit 1
PC: Principle component
PCA: PRINCIPLE Components Analysis
ROH: Runs of homozygosity
*SMG6*: Nonsense Mediated MRNA Decay Factor
SNP: Single-nucleotide polymorphism
*TYRP1*: Tyrosinase related protein 1
