# Supplementary material for: Genomic data illuminates demography, genetic structure and selection of a popular dog breed
Source: BMC Genomics. 2017 Aug 14;18:609. doi: 10.1186/s12864-017-3933-x (PMC5557481; doi:10.1186/s12864-017-3933-x)
Supplement: Supplementary file 1 — Questionnaire used to determine phenotypic and lifestyle information. (PDF 394 kb) [file 12864_2017_3933_MOESM1_ESM.pdf]

12837

Labrador Retriever Hip Dysplasia Study Questionnaire

INSTRUCTIONS:

- Please answer **ALL** questions using a **BLACK PEN**.
- Some questions will provide a space in which you can write your answer.
- Other questions provide answer boxes and require you to mark with a cross either ONE answer or ALL THAT APPLY. **Please indicate your response by placing an "X " in the box that corresponds to your choice. For example** ☒
- To answer the questions regarding girth, height and length of your dog you will need a tape measure.
- Please return the completed questionnaire to us in the envelope provided.

DATA PROTECTION AND CONFIDENTIALITY

Your personal information and the identity of you and your dog will be kept **STRICTLY CONFIDENTIAL** at all times. All data collected from this study will be **ANONYMISED** prior to analysis. The anonymised data may be made publically available at the end of this study in accordance with BBSRC data sharing policies. Return of this document will be taken as consent to use the data you have provided in this study and for future research to improve the welfare of animals.

ARE you willing for us to contact you and send you a mouth swab for you to collect DNA from your dog? ☐ **Yes** ☐ **No**

ARE you willing to complete an additional questionnaire about this dog's behaviour? ☐ **Yes** ☐ **No**

Section A. Owner details

1. Title: Initial: Surname:

2. Address:

3. Postcode:

4. Telephone number:

5. Email address:

FOR OFFICE USE ONLY. FID: ☐☐☐☐☐☐ DID: ☐☐☐☐☐☐ OID: ☐☐☐☐☐☐

12837

Section B: Dog details

6. Breed: Labrador Retriever .

7. Registered name of dog:

8. Kennel Club registration number:

9. Dam's registered name:

10. Sire's registered name:

11. Date of birth:

12. Gender:

13. Coat colour:

14. This dog's pet name: ____________________

15. How old was this dog when you acquired him/her? ____________________

16. In which country was this dog born? | | | | | | | | | | | | | | | | | | | | |

17. Has this dog been neutered? (spayed or castrated) ☐ Yes ☐ No ☐ Don't know

Section C: Activities

18. Is this dog intended for breeding, either now or in the future? ☐ Yes ☐ No

19. Have you bred from this dog in the past? ☐ Yes ☐ No

20. Is this dog a working dog? *Please mark all that apply.*

☐ No - This dog is a pet ☐ Yes - Sniffer Dog

☐ Yes - Gundog ☐ Yes - Hearing Dog

☐ Yes - Guide Dog ☐ Yes - Dog for the Disabled

☐ Yes - Other (please specify): ____________________

12837

21. In which activities does this dog participate? *Please mark all that apply.*

☐ None ☐ Field Trials/Working Tests

☐ Agility Trials ☐ Obedience Trials

☐ Showing ☐ Other - please specify: ______________________

Section D: Exercise and lifestyle

22. In **spring and summer** where does this dog spend the majority of its time? *Please mark only one option.*

☐ In the house ☐ Outside in a kennel/run/garden

☐ Other - please specify: ______________________

23. In **spring and summer** approximately how many hours a day is this dog outside in the open air? (ie not inside the house or kennel)

☐☐ hours

24. In **autumn and winter** where does this dog spend the majority of its time? *Please mark only one option.*

☐ In the house ☐ Outside in a kennel/run/garden

☐ Other - please specify: ______________________

25. In **autumn and winter** approximately how many hours a day is this dog outside in the open air? (i.e. not in the house or kennel)

☐☐ hours

26. How often do you exercise this dog on average? *(*exercise includes all physical activity including training, working and walking.) *Please mark only one option.*

☐ Less than once a day ☐ Once a day ☐ Twice a day ☐ More than twice a day

27. What is the total length of time this dog spends exercising per day on average? *Please mark only one option.*

☐ Up to 1 hour ☐ 1-2 hours ☐ 2-4 hours ☐ More than 4 hours

28. What percentage of the total exercise period is spent unrestricted (off the lead) whilst you are walking, training or working this dog?

☐☐☐ %

12837

Section E: Feeding and body condition

29. What do you feed this dog? *Please mark all that apply.*

☐ Home-cooked food ☐ Tinned food ☐ Dry food

☐ Other - please specify: ____________________

30. Do you feed this dog extras? *Please mark all that apply.*

☐ None ☐ Dog treats ☐ Table scraps

☐ Other - please specify: ____________________

31. Do you add any of the following supplements to this dog's diet? *Please mark all that apply.*

☐ None ☐ Cod liver oil ☐ Glucosamine ☐ Chondroitin

☐ Other - please specify: ____________________

32. What is this dog's current weight?

☐☐☐ kg or ☐☐☐ lb

Please note it is not advisable for you to attempt to lift a Labrador Retriever as it could be hazardous. Your own vet may allow you to weigh your dog using their walk on scales. We would be very grateful if you could provide us with the current weight of your dog, however we are aware that this will not be possible for everyone.

Please refer to the guidelines on the back page (page 8) to assist you with answering the following 3 questions.

33. What is this dog's height at withers? (highest point at shoulder)

☐☐☐ cm or ☐☐☐ in

34. What is this dog's length?

☐☐☐ cm or ☐☐☐ in

35. What is this dog's girth?

☐☐☐ cm or ☐☐☐ in

12837

36. Which of these outlines most resembles this dog's shape?

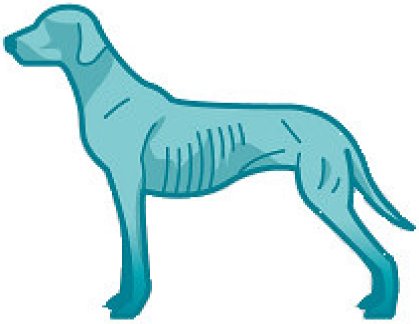

☐ 1

- Ribs, spine and hip bones are easily seen (in short haired dogs)
- Obvious loss of muscle bulk
- No fat can be felt under the skin

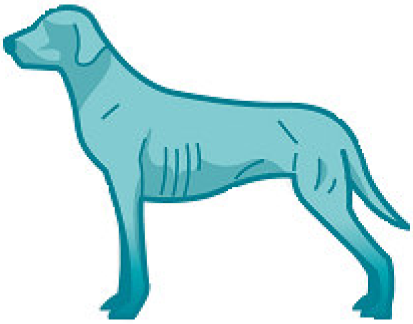

☐ 2

- Ribs, spine and hip bones easily seen
- Obvious waist and abdominal tuck
- Very little fat can be felt under the skin

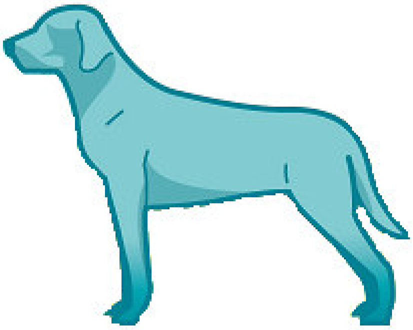

☐ 3

- Ribs, spine and hip bones easily felt
- Visible waist with an abdominal tuck
- A small amount of fat can be felt

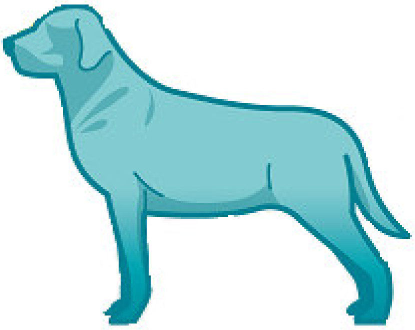

☐ 4

- Ribs, spine and hip bones are hard to feel
- Waist barely visible with a broad back
- Layer of fat on belly and at the base of tail

Pictures and descriptions courtesy of the PFMA.

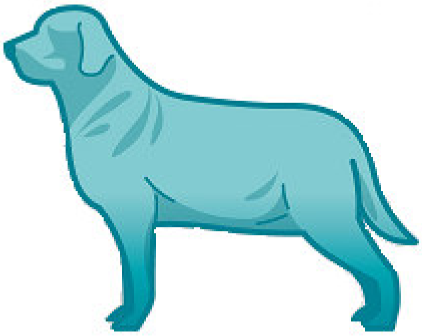

☐ 5

- Ribs, spine and hips extremely difficult to feel under a thick layer of fat
- No waist can be seen and belly may droop significantly
- Heavy fat pads on lower back and at base of tail

Section F: Health

37. Is this dog taking any regular medication prescribed by a vet to treat a diagnosed condition? (other than routine vaccinations or treatment for worms/fleas)

☐ No

☐ Yes - please specify condition(s): ____________________

- please specify medication(s): ____________________

12837

38. Has this dog ever had any disease or veterinary conditions?

☐ Yes - please complete the table on the next page as fully as possible.

☐ No - please go to section G.

We are aware you may not have all of the information we are asking for so please use the 'Other - please specify' sections to tell us what you do know.

**Disease condition**		**Additional information including medication**
Bone, muscle or joint disease	☐ Yes ☐ No	
Arthritis of the hip	☐ Yes ☐ No ☐ Don't know	
of the elbow	☐ Yes ☐ No ☐ Don't know	
of another joint	☐ Yes ☐ No ☐ Don't know	Joint: ______
Osteochondritis dissecans (OCD) of the elbow	☐ Yes ☐ No ☐ Don't know	
of the stifle (knee)	☐ Yes ☐ No ☐ Don't know	
of the shoulder	☐ Yes ☐ No ☐ Don't know	
of the hock (ankle)	☐ Yes ☐ No ☐ Don't know	
Cruciate ligament rupture (CLR)	☐ Yes ☐ No ☐ Don't know	
Patellar luxation	☐ Yes ☐ No ☐ Don't know	
Centronuclear myopathy (CNM)	☐ Yes ☐ No ☐ Don't know	
Other - please specify:	______	______
Eye disease	☐ Yes ☐ No	
Progressive retinal atrophy (PRA)	☐ Yes ☐ No ☐ Don't know	
Retinal dysplasia/oculo-skeletal dysplasia (RD/OSD)	☐ Yes ☐ No ☐ Don't know	
Cataract	☐ Yes ☐ No ☐ Don't know	
Corneal dystrophy	☐ Yes ☐ No ☐ Don't know	
Other - please specify:	______	______
Heart disease	☐ Yes ☐ No	
Heart murmur	☐ Yes ☐ No ☐ Don't know	
Mitral valve disease	☐ Yes ☐ No ☐ Don't know	
Tricuspid valve dysplasia	☐ Yes ☐ No ☐ Don't know	
Pulmonic stenosis	☐ Yes ☐ No ☐ Don't know	
Third degree heart block	☐ Yes ☐ No ☐ Don't know	
Pericardial effusion	☐ Yes ☐ No ☐ Don't know	
Other - please specify:	______	______

12837

Disease condition		Additional information including medication
Allergic skin disease	☐ Yes ☐ No	
Atopy (environmental allergy)	☐ Yes ☐ No ☐ Don't know	
Flea allergy	☐ Yes ☐ No ☐ Don't know	
Food allergy	☐ Yes ☐ No ☐ Don't know	
Other - please specify:	______	______
Cancer	☐ Yes ☐ No	
Has this dog had ANY cancerous tumours or growths?	☐ Yes ☐ No ☐ Don't know	If YES please give any further information you have: ______
Lymphoma	☐ Yes ☐ No ☐ Don't know	
Mast cell tumour	☐ Yes ☐ No ☐ Don't know	
Histiocytoma	☐ Yes ☐ No ☐ Don't know	
Melanoma	☐ Yes ☐ No ☐ Don't know	
Other - please specify type/location	______	______
Other		
Exercise-induced collapse (EIC)	☐ Yes ☐ No ☐ Don't know	
Epilepsy	☐ Yes ☐ No ☐ Don't know	
Laryngeal paralysis	☐ Yes ☐ No ☐ Don't know	
Bloat or gastric torsion (GDV)	☐ Yes ☐ No ☐ Don't know	
Deafness	☐ Yes ☐ No ☐ Don't know	Has this dog been BAER hearing tested? ☐ Yes ☐ No
Diabetes mellitus	☐ Yes ☐ No ☐ Don't know	
Hypothyroidism	☐ Yes ☐ No ☐ Don't know	
Cryptorchidism (undescended testicle)	☐ Yes ☐ No ☐ Don't know	
Other - please specify:	______	______

Section G: Comments

This space is for you to make any additional comments that you may have on the questionnaire, this study or specific issues that you feel are important. Your input is welcomed.

______

______

______

______

How to measure your Labrador Retriever.

Things you will need beforehand:

- A flexible measuring item i.e. tape measure or a leash or string which can be marked and measured against a yard stick
- Paper and a pen to write down the measurements
- It can be helpful to have a second person with you especially if your dog tends to squirm. A second person is invaluable when measuring your dog's height.

It is advisable to measure your dog on a flat non-slip surface.

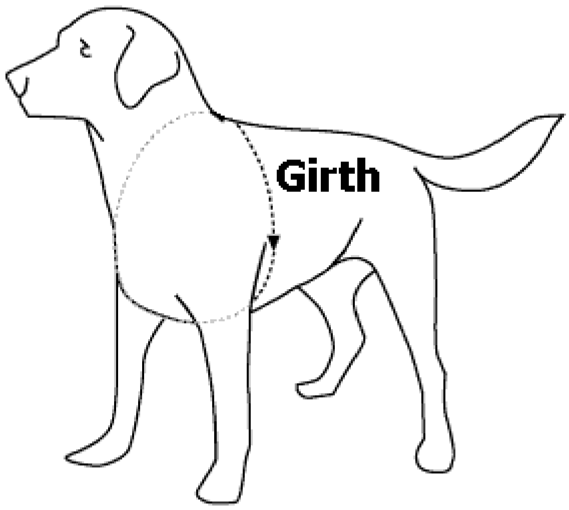

To measure your Labrador's girth:

Stand over your dog and place one end of the tape measure on his spine, just above the widest part of his rib cage. With your other hand, wrap the tape measure behind his front legs and around his body, going underneath the widest part of his rib cage. Place this end next to the beginning of the tape. Put your thumb on the number that meets up with the beginning of the tape measure. The number is the girth measurement.

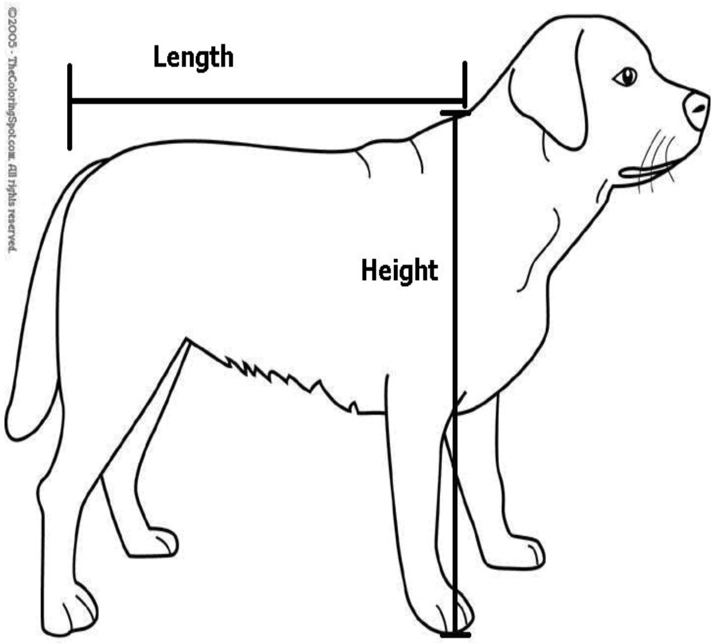

Above picture courtesy of thecoloringspot.com

To measure your Labrador's height:

You will require your helper to stand to one side of the dog running the tape measure from the floor up toward the dogs shoulders (*withers)* Using the edge of a pen or pencil you can identify where the dogs withers reach on the tape measure.

To measure your Labrador's length:

Stand over your dog and place one end of the tape measure immediately behind his neck at the top of the dogs shoulder blades (*withers)* to the start of the dog's tail.

Weighing your Labrador:

Please note it is not advisable for you to attempt to lift a Labrador Retriever as it could be hazardous.

Your own vet may allow you to weigh your dog using their walk on scales. We would be very grateful if you could provide us with the current weight of your dog, however we are aware that this will not be possible for everyone.
