# Supplementary figures and images for: Genomic data illuminates demography, genetic structure and selection of a popular dog breed
Source: BMC Genomics. 2017 Aug 14;18:609. doi: 10.1186/s12864-017-3933-x (PMC5557481; doi:10.1186/s12864-017-3933-x)

## Slide 1
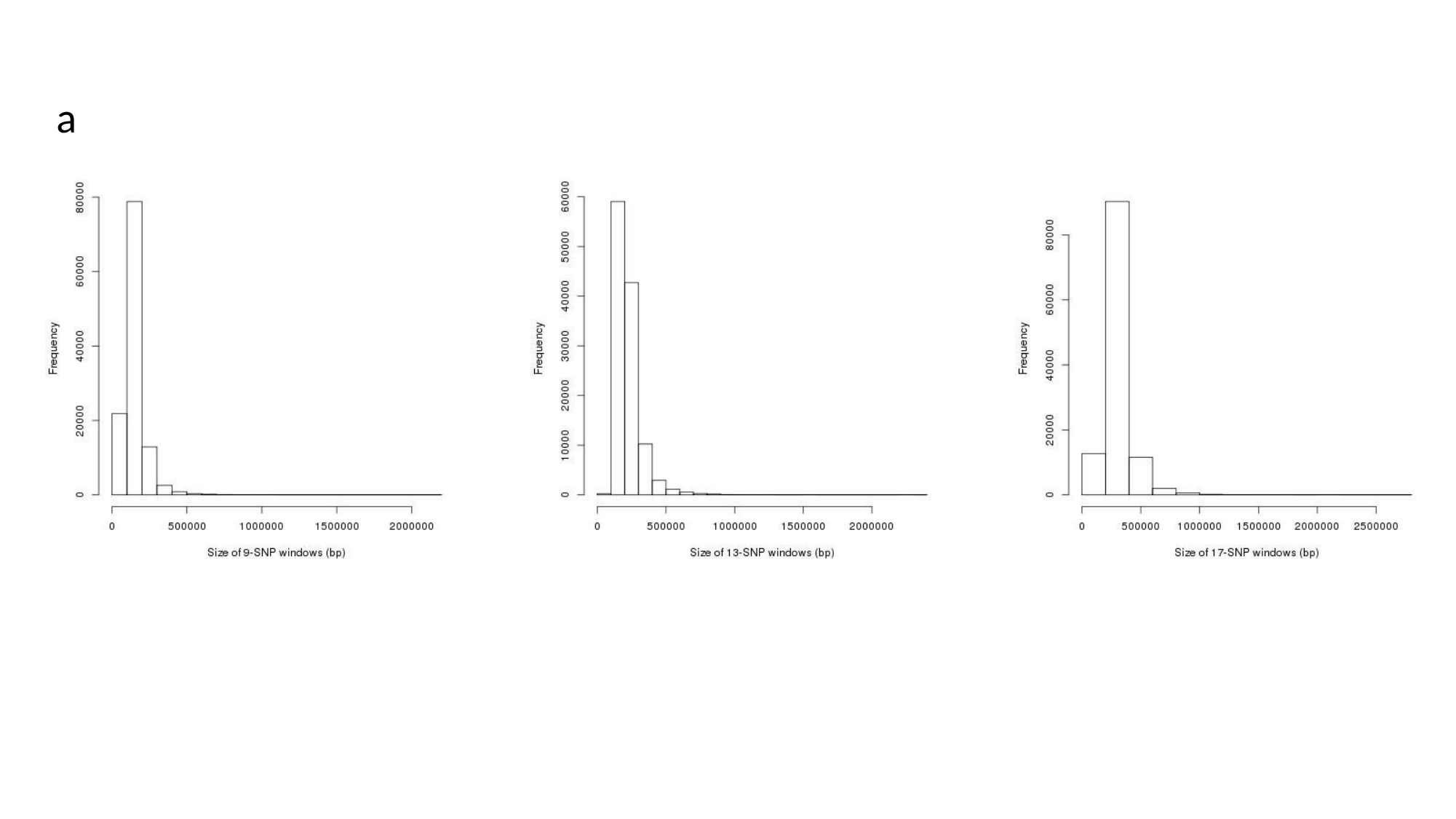

a

## Slide 2
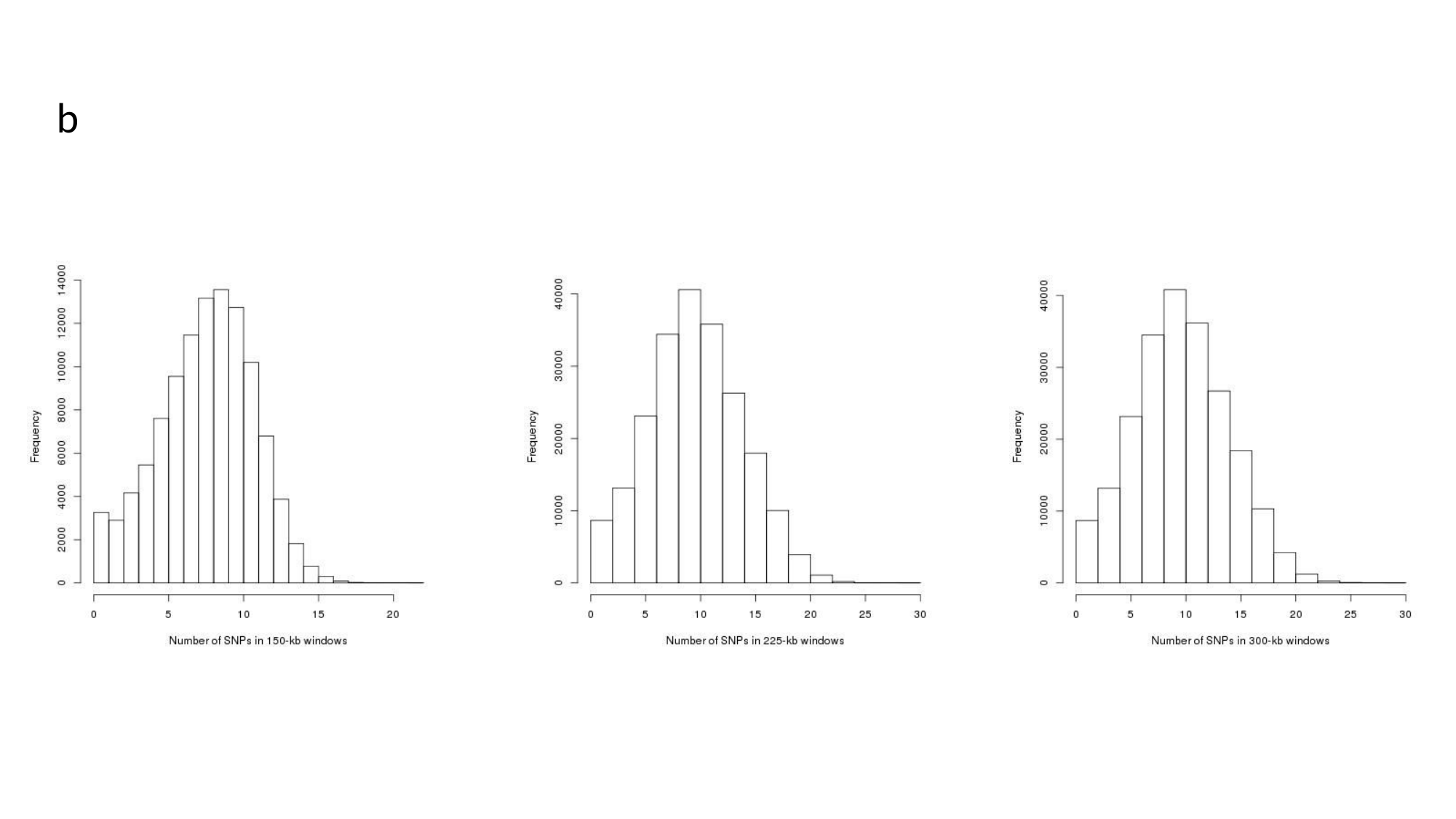

b

Supplement: Supplementary file 2 — a. Distributions of physical size for windows of fixed number of SNPs (9, 13, 17). b. Distributions of number of SNPs for windows of fixed physical size (150Kb, 225Kb, 300Kb; approximate genome-wide average sizes of 9-SNP, 13-SNP and 17-SNP windows). (PPTX 117 kb) [file 12864_2017_3933_MOESM2_ESM.pptx]
